# Primary data on symptom burden and quality of life among elderly patients at risk of dying during unplanned admissions to an NHS hospital: a cohort study using EuroQoL and the integrated palliative care outcome scale

**DOI:** 10.1186/s12904-024-01384-9

**Published:** 2024-02-20

**Authors:** Bridget M. Johnston, Mary Miller, Charles Normand, Magnolia Cardona, Peter May, Aoife C. Lowney

**Affiliations:** 1 Centre for Health Policy and Management, Trinity College Dublin, University of Dublin, 3-4 Foster Place, Dublin 2, Dublin, Ireland; 2 Department of Palliative Care, Oxford University Hospitals NHS Foundation Trust, Oxford, England; 3 Nuffield Department of Medicine, Oxford University, Oxford, England; 4 Cicely Saunders Institute of Palliative Care, Policy and Rehabilitation, King’s College London, London, England; 5 Faculty of Health and Behavioural Sciences, School of Psychology, The University of Queensland, Brisbane, Australia; 6 Institute for Evidence Based Healthcare, Bond University, Gold Coast, Australia; 7 Department of Palliative Care, Marymount University Hospital and Hospice and Cork University Hospital, Cork, Ireland; 8 University College Cork, Cork, Ireland

**Keywords:** Palliative care, Symptom burden, Older persons, Hospital care, Integrated palliative care outcomes scale, EQ-5D-5L, Quality of life, Health economics

## Abstract

**Background:**

Older people account heavily for palliative care needs at the population level and are growing in number as the population ages. There is relatively little high-quality data on symptom burden and quality of life, since these data are not routinely collected, and this group are under-recruited in primary research. It is unclear which measurement tools are best suited to capture burdens and experience.

**Methods:**

We recruited a cohort of 221 patients aged 75 + years with poor prognosis who had an unplanned admission via the emergency department in a large urban hospital in England between 2019 and 2020. Risk of dying was assessed using the CriSTAL tool. We collected primary data and combined these with routine health records. Baseline clinical data and patient reported quality of life outcomes were collected on admission and reassessed within the first 72 h of presentation using two established tools: EQ-5D-5 L, EQ-VAS and the Integrated Palliative Outcomes Scale (IPOS).

**Results:**

Completion rate was 68% (*n* = 151) and 33.1% were known to have died during admission or within 6 months post-discharge. The vast majority (84.8%) reported severe difficulties with at least one dimension of EQ-5D-5 L at baseline and improvements in EQ-VAS observed at reassessment in 51.7%. The baseline IPOS revealed 78.2% of patients rating seven or more items as moderate, severe or overwhelming, but a significant reduction (-3.6, *p* < 0.001) in overall physical symptom severity and prevalence was also apparent. No significant differences were noted in emotional symptoms or changes in communication/practical issues. IPOS total score at follow up was positively associated with age, having comorbidities (Charlson index score > = 1) and negatively associated with baseline IPOS and CriSTAL scores.

**Conclusion:**

Older people with poor prognosis admitted to hospital have very high symptom burden compared to population norms, though some improvement following assessment was observed on all measures. These data provide valuable descriptive information on quality of life among a priority population in practice and policy and can be used in future research to identify suitable interventions and model their effects.

**Supplementary Information:**

The online version contains supplementary material available at 10.1186/s12904-024-01384-9.

## Introduction

Worldwide, populations are ageing and the number of older people living with serious illnesses and complex needs is increasing [1–3]. Use of emergency and urgent care departments (EDs) by older people is also on the rise, especially towards the end of life. In one study, over one-half of decedents aged 65 years or older during a 15-year period were found to have visited the ED in the last month of life, and 75% visited during the last six months, many repeatedly [4]. Indeed, a quarter of patients in acute hospitals in the UK are thought to be in the last year of life [5]. Older patients account disproportionately for palliative care needs at the population level and are driving rapid increases in need [1], but high-quality data on symptom burden are seldom collected routinely and this population are underrepresented in trials and other primary studies [6].

There is increasing recognition that applying palliative care principles earlier in the disease trajectory, according to peoples’ needs, improves quality of life for people with life-limiting illness and their loved ones and can reduce pressure on health and social care systems [7–10]. The identification of patients with life-limiting illness can trigger important advance care planning conversations – a priority for health and social care agencies in England [11]. Adhering to preferences, avoiding inappropriate investigations and non-beneficial treatments and providing quality end of life care for elderly patients at risk of dying in hospital during unplanned admissions is challenging, given that most of what is reported in the literature related to this population focuses on service-related metrics (e.g., mortality and admissions) as opposed to patient-centred metrics [12]. There is also ongoing disagreement on the best tools for measuring quality of life in palliative care populations, given an inherent trade-off between generic measures such as EuroQol tools, which maximise comparability with other populations and interventions, and context-specific measures such as the Integrated Palliative Care Outcomes Scale (IPOS), which maximise sensitivity to the circumstances and priorities in the relevant population [13–15]. This lack of consensus has contributed to a near total absence of economic evaluations of palliative care [16, 17].

The aim of this paper is to report primary data using describing symptom burden and factors related to observed variation among patients 75 and older, identified to be at risk of dying, during an unplanned hospital admission. This information can contribute to three areas of policy and service development. First, evidence about the needs and symptom burden experienced by this population can be used to better understand the circumstances of this under-studied population. Second, arising data can support efforts to identify interventions and services most likely to have a significant impact on health and well-being, such as proactive palliative care. Third, data from multiple patient-reported outcome measures can be used to advance the sparse economic evidence base in palliative care, both through modelling exercises and comparative assessment of different quality of life tools.

## Methods

### Design and context

This study was initially designed as a prospective cohort study of early proactive palliative care for patients coming through the emergency department to acute medical wards and who were approaching end-of- life. The aims were to: (1) describe patient symptom burden and health-related quality of life and caregiver strain; and (2) determine cost-effectiveness of proactive palliative care input. The study was conducted in two phases between January 2019 and December 2020, with active recruitment for 18 months during this period. Recruitment to phase 1 of the study was conducted from January to December 2019, gathering data and providing usual care. Usual care was defined as a review triggered by the patient’s consultant and treating team requesting specialist palliative care input. Recruitment to phase 2 began in January 2020 and was halted by the SARS-CoV-2 pandemic in early March 2020, as all non-coronavirus research was paused in the Oxford hospitals. The study reopened in September 2020, closing in December 2020 on recognition that ED and medical admissions had substantially reconfigured and patients recruiting to phase 2 were different to those in phase 1. Therefore, what is instead presented in this paper is pooled analysis for individuals recruited to the study describing their symptom burden and health-related quality of life trajectory over the course of a 24 to 72-hour period, in keeping with the stipulated timeframe in the originally planned study.

### Ethics

The study received ethical approval from the NHS Health Research Authority (England) Research Ethics Committee in Oxford, England (18/SC/0488). In addition, NHS sponsorship (Oxford University Hospital NHS Foundation Trust) was obtained.

### Study recruitment

Recruitment took place over 18 months between January 2019 and December 2020.

#### Screening

Patients over the age of 75 accepted for admission under Acute General Medicine having first presented via the ‘front door’ of the hospital (emergency department or emergency assessment unit, John Radcliffe Hospital, Oxford University Hospitals Trust) during office hours were screened by the emergency department research team using the study inclusion criteria. This team was embedded at the front door, working five days per week. The supervising team could refer for a palliative care consultation if needed.

#### Inclusion criteria

- patient (or their consultee) was able to understand the information given about the study and give consent;
- patient ≥ 75 years;
- decision to admit to care of inpatient medical team;
- CRiSTAL score ≥ 6.

#### Exclusion criteria

- too unwell to take part as voiced by the patient or determined by clinical team;
- elective admission.

A CRiSTAL threshold of 6 or higher was chosen based on previous Danish and Irish cohort studies indicating that patients who had a short-term death were more likely to have their score in the 6 or higher risk levels [18, 19]. The tool was designed based on objective criteria available at the point of care, including the presence of advanced chronic illness, frailty parameters, history of hospital/ICU admission, physiological deterioration criteria, and nursing home residency status.

#### Consent

Individuals meeting the eligibility criteria were approached and offered verbal and written information about the study by the research team. The team with primary responsibility for the patient identified a consultee/carer for those individuals who lacked capacity to provide informed consent. Individuals were given sufficient time to consider whether they would like to participate and provided written or verbal consent if wishing to take part.

### Data collection

Individuals enrolled in the study had their symptom burden and quality of life captured at admission and at a second time point between 24 and 72 h later. The 72-hour interval was guided by practical aspects of clinical palliative care, whereby timely assessment is essential to ensure patients symptoms and concerns are managed as quickly as possible. Early reassessment also reduced the potential for attrition due to death, clinical deterioration or discharge to another care setting. Participants completed outcome measure questionnaires face-to-face, with help from a research nurse where needed. Reasons for non-participation in baseline or reassessment interviews werenot systematically captured.

Routine demographic and health record data were also collected. Hospital and demographic data and utilisation data were collected from patient paper records and electronic health records (EHR). Data collected from the Cerner Millennium EHR via an information request to the IT department included: admission and discharge dates and times, dates of birth and death, gender, ethnic group, religion, number of drug administrations during admission, DNAR status, number of times a patient moved ward during their hospital admission, ICD 10 codes and procedure codes. Individuals were followed via their EHR, to determine readmission rates and survival, to the point of death or until six months after the final participant was recruited to the study (occurring September 2021). The individual’s name and NHS number were cross referenced against the palliative care departmental database (iCare) to determine if patients were known to the service prior to their enrolment in the study.

Study data were anonymised and stored on NHS password protected servers or in secure filing cabinets. Data shared for analysis were not identifiable in accordance with data sharing agreements in place with research collaborators.

#### Questionnaires

Health-related quality of life was captured using two established, previously published, measures: the EQ-5D-5 L [20] and the Integrated Palliative Outcomes Scale (IPOS) [21]. EQ-5D-5 L contains five dimensions which measure health status: mobility, self-care, usual activity, pain/discomfort, and anxiety/depression. Each of these dimensions is evaluated by five levels (1 to 5) ranging from no problems [1] to extreme problems [5]. The health ratings for each dimension can be converted to a single summary number, utility index score, using validated EQ-5D-5 L value sets, with higher scores indicating higher health-related quality of life. These value sets can allow for comparison to population norms and preferences. EQ-5D includes a visual analogue scale, EQ-VAS, a continuous response scale ranging from 0 (worst imaginable health state) to 100 (best imaginable health state), to record self-rated health at the time of assessment [22]. Similar to EQ-5D-5 L, increased scores in the EQ-VAS indicate better health.

IPOS captures information on 17 items related to common physical symptoms, patient/carer anxiety, emotional well-being, information received and practical concerns over three days that precede the assessment [21]. Each item is evaluated on a five-point Likert scale, ranging from 0 (not at all) to 4 (overwhelming/all the time). Previous research has identified three subscales within IPOS: physical symptoms (10 items); emotional symptoms (4 items); and communication/practical issues (3 items). Increased IPOS scores represent rising symptom burden and reduced health-related quality of life.

### Analysis

All analyses were completed using Stata 17. Participants were included in the full analysis if they had completed both the baseline and follow-up interview.

Utility index scores from the EQ-5D-5 L were calculated using a UK-specific value set [23]. EQ-VAS scores were recorded directly from the scale.

The prevalence, mean, standard deviations of each item, and the total score for all items and subscales within IPOS were calculated. Prevalence was defined as any IPOS symptoms/concerns specified as moderate, severe or overwhelming. Total IPOS score was calculated as the total of all items and the total of each IPOS subscale was scored using the relevant items [21].

Correlation between the three outcome measures (EQ-5D-5 L, EQ-VAS, and IPOS) was evaluated with Spearman’s rank correlation coefficient (ρ). A weak correlation was defined as ρ < 0.30, a moderate correlation as 0.30 ≥ ρ < 0.50 and a strong correlation as ρ ≥ 0.50.

Multiple linear regression models with heteroskedasticity-robust standard errors were estimated to examine the association between patient clinical and demographic characteristics, EQ-5D-5 L utility index, EQ-VAS and IPOS total scores. Variable selection was informed by relevant literature and data availability. We modelled associations between characteristics and reassessment scores rather than differences between the two assessment periods. This is because baseline values are generally negatively correlated with change, meaning people with low scores at baseline tend to improve more than people with higher scores [24].

## Results

A total of 221 participants were recruited to the study (See Additional File 1). Characteristics of the 151 participants (68%) who completed both a baseline and reassessment interview are reported in Table 1. The median age was 84 years, 36.4% were known to the departmental palliative care service prior to admission and 66.2% had a Charlson Index Score of 2 or more. Most participants (86.8%) had a non-cancer diagnosis.

**Table 1:** Sample characteristics

Variable	Value	Missing data
**Gender: Female (%)**	75 (49.7%)	2 (1.3%)
**Age**: Median (IQR)	84 (80–88)	0
**DNACPR in place**	106 (70.6%)	0
**Marital status**		11 (7.3%)
Single	8 (5.3%)	
Married	54 (35.7%)	
Divorced	7 (4.6%)	
Widowed	71 (47.0%)	
**CRiSTAL score**		1 (0.7%)
6	59 (39.1%)	
7	50 (33.1%)	
8	20 (13.3%)	
9	16 (10.6%)	
10	4 (2.7%)	
11	1 (0.7%)	
**Charlson Index score**		0
0	19 (12.6%)	
1	32 (21.2%)	
2 or higher	100 (66.2%)	
**Cancer**	20 (13.2%)	
**Deprivation**		0
2	2 (1.4%)	
3	3 (2%)	
4	3 (2%)	
5	8 (5.4%)	
6	19 (12.9%)	
7	17 (11.5%)	
8	40 (27%)	
9	26 (17.6%)	
10	30 (20.3%)	
Known to department PC*	55 (36.4%)	0
**Length of stay**: median days (SD)	7 [9] Range: 1–67	0
**Died during index admission**	13 (8.6%)	0
**Time from index admission to death**: median days (SD)	67 (171.7)	0
**Readmissions in six months following the index admission**: Mean (Range)	0.69 (0–8)	0
**ED reattendance in following six months**	11 (7.3%)	0
Died post discharge and within 6 months of enrolment**	37 (24.5%)	0

*Known to PC: Had an interaction with departmental palliative care service prior to this admission

**Total deaths between admission and 6 months follow up = 50 (33.3% of study population)

### Measuring health-related quality of life using EQ-5D-5 L

Details of EQ-5D-5 L utility index and EQ-VAS scores at baseline and reassessment are provided in Table 2. Mean (SD) EQ-5D-5 L index value at baseline was 0.36 (0.261) and ranged from − 0.226 to 1, with two participants (1.3%) describing perfect health and 16 participants (10.6%) with a negative utility index (indicating health state worse than death). Mean (SD) EQ-5D-5 L index value at reassessment was 0.44, ranging from − 0.226 to 1. Only one participant (0.66%) now reported perfect health, and 9 (5.9%) reported a negative utility index. The difference in utility index values between baseline and reassessment represents an improvement of 0.086 (*p* < 0.001). Mean (SD) EQ-VAS at baseline was 45.1 (24.5), increasing to 49.9 (22.8) at reassessment.

**Table 2:** Summary of EQ-5D-5 L utility index and EQ-VAS scores at baseline and reassessment

	Baseline	Reassessment	Difference (pts)	***p***-value
EQ-5D-5 L utility index value (SD)	0.355 (0.275)	0.441 (0.285)	0.086	0.000
Total EQ-5D-5 L dimensions rated as severe or extreme				
0	23 (15.2%)	38 (25.2%)		
1	25 (16.6%)	25 (16.6%)		
2	27 (17.9%)	26 (17.2%)		
3	52 (34.4%)	45 (29.8%)		
4	19 (12.6%)	11 (7.3%)		
5	5 (3.3%)	6 (4%)		
EQ-VAS (SD)	45.1 (24.5)	49.9 (22.8)	4.8	0.025

**Fig. 1:**
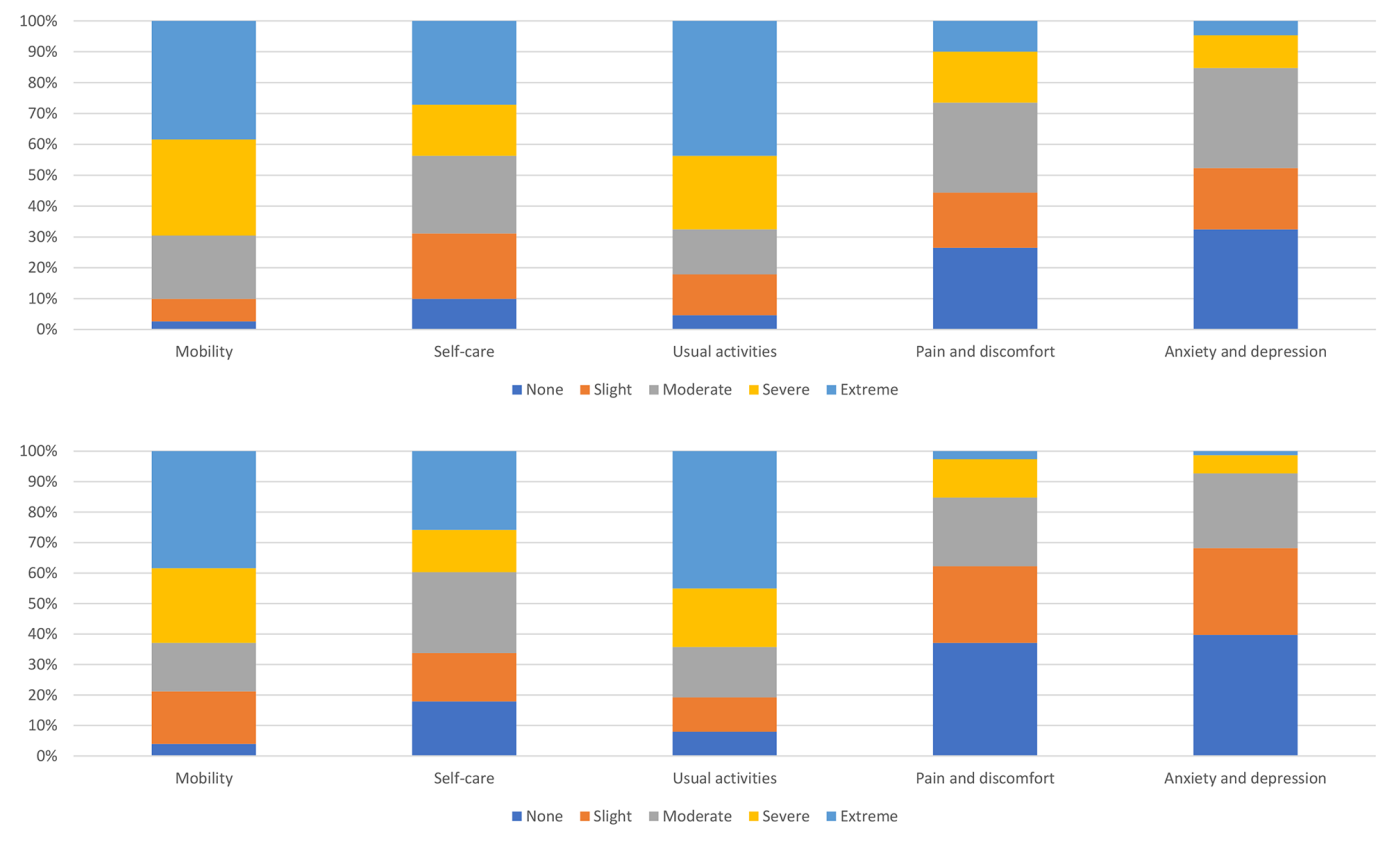
Distribution of responses by level of severity for EQ-5D-5 L dimensions at baseline and reassessment

Most participants (84.8%) reported severe or extreme difficulties with at least one EQ-5D-5 L dimension at baseline (Table 2). Approximately half (50.3%) of participants reported severe or extreme difficulties with three or more dimensions. The dimension associated with greatest impact on health-related quality of life for patients at baseline were mobility and usual activities, with 69.5% of patients reported severe or extreme problems for mobility and 67.5% for usual activities (Fig. 1). A significant proportion (43.7%) also experienced severe or extreme problems for the self-care dimension.

At reassessment, the proportion reporting severe or extreme difficulties with at least one EQ-5D-5 L dimension at decreased to 74.8%. Similarly, the number reporting severe or extreme difficulties with three or more dimensions also fell to 41.4% (Table 2). Improvements between baseline and reassessment were observed across all five dimensions, including ‘pain and discomfort’ and ‘anxiety and depression’ (Fig. 1).

The largest reduction in problems experienced between baseline and reassessment was for the ‘pain and discomfort’ dimension (47%) (Table 3). While problems associated with the ‘anxiety and depression’ dimension also decreased for a large proportion of participants (39.7%), a similar proportion reported no change at all. About one-third reported decreases in challenges related to mobility, self-care and usual activities; however, the proportion reporting their symptoms remained unchanged was higher for these dimensions. Overall, 51.7% reported an increase in their health using the EQ-VAS.

**Table 3:** **Changes in EQ-5D-5 L dimensions and EQ**-**VAS scores between baseline and reassessment**

Dimension	Decreased	Unchanged	Increased
Mobility	31.8	48.3	19.9
Self-care	35.1	41.7	23.2
Usual activities	33.1	40.4	26.5
Pain and discomfort	47.0	37.1	15.9
Anxiety and depression	39.7	39.7	20.5
**EQ VAS**			
Total score	33.1	15.2	51.7

### Measuring health-related quality of life using IPOS

Table 4 shows IPOS total and subscale scores at baseline and reassessment. Mean (SD) IPOS total score at baseline was 27.8 (9.2) and ranged from 3 to 52. Just over three-quarters of participants (78.2%) rated seven or more items as moderate, severe or overwhelming.

**Table 4:** Summary of IPOS total and subscale scores at baseline and reassessment

	Baseline	Reassessment	Difference (pts)	***p***-value
IPOS Total (SD)	27.8 (9.2)	24.2 (9.0)	3.6	< 0.001
IPOS Physical symptoms: 10 items (SD)	17.2 (6.6)	14.5 (6.3)	2.7	< 0.001
IPOS Emotional symptoms: 4 items (SD)	7.4 (3.6)	6.8 (3.7)	0.6	0.072
IPOS Communication/ practical issues: 3 items (SD)	3.1 (2.5)	2.8 (2.5)	0.3	0.213
Total IPOS items rated as moderate, severe or extreme				
0	1 (0.7%)	0		
1–3	6 (3.9%)	17 (11.3%)		
4–6	25 (16.6%)	37 (24.5%)		
7–9	57 (37.8%)	56 (37.1%)		
10–13	56 (37.1%)	37 (24.5%)		
14–17	5 (3.3%)	4 (2.6%)		

Mean (SD) IPOS total score at reassessment was 24.2 (9.0), ranging from 3 to 48, with 64.2% rating seven or more items as moderate, severe or overwhelming. The difference in total score between baseline and reassessment was − 3.6, representing a reduction in overall symptom severity and prevalence of practical issues among participants (*p* < 0.001). All subscale scores decreased between baseline and reassessment; however, the differences in IPOS subscales ‘emotional symptoms’ and ‘communication/practical issues’ were not statistically significant.

The most troublesome physical symptom reported at baseline using IPOS was poor mobility (85.4%), in keeping with the EQ-5D-5 L scores (Table 5). Other frequently reported symptoms included weakness (79.5%), sore or dry mouth (64%), shortness of breath and drowsiness (61.6%), poor appetite (59.6%) and pain (51.7%). A breakdown of ratings for each item at baseline and reassessment are provided in Additional File 2.

**Table 5:** IPOS items at baseline and reassessment

Subscale	Prevalence (%)		Mean (SD)		Difference	***p***-value
	Baseline	Reassessment	Baseline	Reassessment		
**Physical Symptoms**						
Poor mobility	85.4	80.8	2.79 (1.13)	2.57 (1.17)	-0.22	0.04
Weakness	79.5	72.9	2.42 (1.16)	2.21 (1.13)	-0.21	0.05
Sore or dry mouth	64.0	58.9	1.93 (1.27)	1.70 (1.13)	-0.23	0.03
Shortness of breath	61.6	46.4	1.98 (1.35)	1.52 (1.24)	-0.46	0.00
Drowsiness	61.6	56.3	1.88 (1.27)	1.72 (1.19)	-0.16	0.20
Poor appetite	59.6	48.4	1.85 (1.31)	1.52 (1.30)	-0.33	0.009
Pain	51.7	46.3	1.60 (1.30)	1.33 (1.20)	-0.27	0.007
Constipation	34.5	32.9	1.04 (1.20)	0.90 (1.15)	-0.14	0.23
Nausea	29.8	20.5	0.92 (1.21)	0.72 (1.02)	-0.20	0.03
Vomiting	25.2	7.3	0.79 (1.45)	0.30 (0.77)	-0.51	0.00
**Emotional symptoms**						
Family anxiety	78.2	75.5	2.50 (1.37)	2.38 (1.34)	-0.12	0.32
Patient anxiety	54.4	49.1	1.62 (1.33)	1.38 (1.27)	-0.24	0.06
Feeling depressed	39.1	35.0	1.25 (1.24)	1.20 (1.29)	-0.05	0.67
Feeling at peace	57.0	49.7	2.03 (1.38)	1.87 (1.29)	-0.16	0.22
**Communication/practical issues**						
Able to share feelings	47.1	43.0	1.57 (1.45)	1.46 (1.48)	-0.11	0.45
Adequate information received	25.2	26.5	1.08 (1.16)	1.03 (1.23)	-0.05	0.66
Practical matters addressed	15.9	9.9	0.50 (1.04)	0.34 (0.78)	-0.16	0.12

The prevalence of all symptoms fell between baseline and reassessment; vomiting, shortness of breath and poor appetite decreasing most. The fall in reported symptoms did not achieve statistical significance for weakness (*p* = 0.52), constipation (*p* = 0.23) and drowsiness (*p* = 0.20).

Within the ‘emotional symptoms’ domain, approximately 78% of patients reported family anxiety at baseline, and 54.4% reported they were personally experiencing anxiety. The prevalence of all four items in this domain fell between baseline and reassessment, though none of the differences were statistically significant.

Only 25.2% of patients described having received adequate information. This was the only symptom or issue where prevalence increased between baseline and reassessment, though this was by a small amount and the change was not statistically significant (*p* = 0.66).

### Factors associated with patterns in symptom burden and trajectories

The correlation between the three outcome measures at baseline was moderate, while strength of the relationship between EQ-5D-5 L utility scores and EQ VAS, and IPOS total score and EQ-VAS was weak at reassessment (see Additional File 3 for further details). IPOS scores were negatively correlated with EQ-VAS and EQ-5D-5 L utility scores at both baseline and reassessment; however, this was expected as IPOS total score increases with growing symptom burden. EQ-5D and EQ-VAS are positively correlated at both baseline and reassessment.

Table 6 reports the results from the multivariate regression analyses of the association between the EQ-5D-5 L index score, the EQ VAS, the IPOS total score and patients’ demographic and clinical characteristics. There was no statistically significant association observed between EQ-5D-5 L index score or EQ VAS and patients’ demographic characteristics; however, baseline EQ-5D-5 L scores were significantly positively associated with follow-up EQ-5D-5 L scores (b = 0.53; 95% CI 0.30–0.76). Similarly, higher baseline EQ-VAS scores were also positively associated with follow up EQ-VAS scores (b = 0.33; 95% CI 0.13–0.53). IPOS total score at follow-up was negatively associated with baseline EQ-5D-5 L scores (b = -7.37; 95% CI -12.9 - -1.74) and CriSTAL scores. Additionally, IPOS total score at follow up was positively associated with baseline IPOS, age, and having comorbidities (Charlson index score > = 1).

**Table 6:** Linear regression analyses of associations between outcome measures and patients’ characteristics

Variable	EQ-5D-5 L index score	EQ-VAS	IPOS total score
Baseline EQ-5D-5 L	0.53 (0.11)***	-5.90 (9.37)	-7.37 (2.84)*
Baseline VAS	-0.00 (0.00)	0.33 (0.10)**	0.05 (0.33)
Baseline IPOS	-0.00 (0.00)	-0.17 (0.24)	0.44 (0.90)***
Gender (ref: male)	0.06 (0.05)	-3.98 (4.54)	0.30 (1.26)
Age	0.00 (0.00)	-0.36 (0.48)	0.28 (0.11)*
Charlson (ref: 0)			
1	0.03 (0.09)	-0.84 (9.42)	7.54 (2.25)**
2	0.07 (0.08)	2.41 (8.43)	4.75 (0.11)**
CRISTAL score	-0.00 (0.20)	-1.47 (2.04)	-1.18 (0.57)*
Known to PMS	-0.02 (0.05)	2.27 (3.90)	2.11 (1.35)
Marital status (ref: single)			
Married	0.01 (0.12)	15.13 (7.89)	-2.17 (2.71)
Divorced	0.12 (0.16)	11.92 (8.78)	-1.21 (4.46)
Widowed	0.00 (0.12)	14.13 (9.23)	-2.56 (2.90)
Constant	0.16 (0.43)	68.97 (51.3)	-6.01 (11.36)

Unstandardized beta-coefficients are reported; robust standard errors in parentheses

***p < 0.001, **p < 0.01, *p < 0.05

## Discussion

This study provides evidence about the patterns of symptom burden among older people at risk of dying during an unscheduled hospital admission via the emergency department or emergency assessment unit and the factors contributing to observed patterns. The findings indicate that a notable proportion of participants were experiencing significant and persistent challenges related to mobility, self-care, weakness and carrying out usual activities but physical symptoms improved in the first 72 h while there was no significant change in emotional symptoms. Patient reported palliative outcomes were positively associated with the presence of comorbidities and age.

### What do we learn about the population and needs?

The mean EQ-5D-5 L utility index scores at both baseline and follow-up (0.355 and 0.441, respectively) were much lower than population norms for the UK among people aged 75 and over (0.661–0.756) [25]. Mean EQ-VAS scores were also much lower at baseline and follow-up (45.1 and 49.9, respectively) than previously reported population norms (0.74). However, this finding is not surprising, given participants were living with serious illness and at risk of dying. While there is significant variation in mean index utility scores reported among previous studies in similar populations [26–28], the overarching trend is that these values are typically much lower than population norms. Our findings can provide a valuable data source for future cost-effectiveness modelling studies evaluating interventions for older people with serious illness, particularly given that older people with high symptom burden are underrepresented in trial data [6].

While EQ-5D can provide a valuable framework for understanding the physical health states experienced by people living with serious illness, it may not capture or be sensitive enough to changes in the specific needs and concerns contributing to quality of life in this population [21, 26, 29]. The IPOS tool captures important information about commonly experienced symptoms and concerns among those with palliative care needs (e.g., poor appetite, sore mouth, nausea and information provision) in addition to caregivers’ wellbeing. Although only a third of participants were known to the hospital’s palliative care consultative service, many of the key patterns observed in this study are surprisingly similar to those previously reported among people accessing specialist palliative care. For example, poor mobility and weakness were the most frequently experienced physical symptoms (prevalence 85% and 80%, respectively). This is in keeping with patterns among a sample of adults receiving specialist palliative care in acute and community settings as reported by Murtagh et al., with 82% experiencing moderate, severe or overwhelming weakness and 77.4% with poor mobility [21]. Additionally, a sore or dry mouth was the third most prevalent symptom at baseline and reassessment (64% and 58.4%, respectively) which is consistent with rates reported among people hospitalised with heart failure [30]. Participants also had similar or higher levels of both physical and emotional symptom burden than among people accessing community-based specialist palliative care services as identified by Nicholson et al. [31].

### Observed trajectories and factors associated with these patterns

Average physical symptom burden appeared to improve between baseline and reassessment. While it was beyond the scope of this study to estimate the minimally important difference (MID) in EQ-5D-5 L and EQ-VAS, the increase in EQ-5D-5 L index score of 0.086 between the two periods is higher the estimated MID threshold for England of 0.037 [32]. It is important to note that MID estimates are sensitive to a range of factors including the computational methods applied, whether patient judgements about improvement are captured and the population or conditions being considered [32]. Future studies seeking to estimate EQ-5D-5 L MID specific to this population should include additional validated outcome measures that would allow for anchor-based methods to be applied in combination with the instrument-defined MID.

IPOS total scores and all subscale scores decreased between the two time points, also suggesting improvements in the prevalence and severity of physical and psychosocial burden. However, it is not possible to determine if the changes in total IPOS, subscale scores and single items observed represent a minimal clinically important change for improvement or deterioration as these values have not been identified [21]. Establishing and validating thresholds for minimal clinically important change is a priority area for future research. It is important to note that our study captures a limited period in care trajectory and interventions to address some dimensions of well-being may require a longer timeframe to deliver improvements. Additionally, IPOS utilises a three-day recall period which may have resulted in overlap between baseline and follow-up assessments among some participants.

We also sought to explore associations between participant characteristics and observed trajectories in health-related quality of life and symptom burden. The multivariate analysis found limited evidence about the factors that influence reported EQ-5D-5 L index utility scores at reassessment. Indeed, the only significant association was a positive and significant correlation between baseline index score and follow-up index score. Similar trends were observed for the EQ-VAS scores. However, there were significant associations between IPOS scores and some individual factors including age, baseline IPOS and EQ-5D scores and the Charlson and CRISTAL scores. The results offer a mixed picture; while IPOS scores at follow-up are significantly and positively associated with Charlson Index, we observed a significant and negative relationship between IPOS and CRiSTAL. These findings should be interpreted with caution as the positive association between IPOS at baseline and follow up might be explained by the potential overlap in recall period for many participants. Unlike EQ-5D-5 L which asks about health status at the time the questionnaire is being completed, IPOS prompts individuals to rate symptoms and issues over the past three days. Further, CRiSTAL scores were used as an inclusion criterion for the study, resulting in limited variation in these data.

### Policy implications

This cohort is older, mainly with non-cancer conditions, yet they are experiencing a symptom burden that is similar, or in some instances higher, to those utilising specialist palliative care services. Research continues to demonstrate that strategies to improve care and outcomes for this population must include systematic and holistic assessment of needs and support for decision making [33]. For example, ED admission triggers to expedite specialist palliative care engagement are associated with a 50–75% reduction in both hospital length of stay and costs when compared against usual palliative consultation practice [34]. However, it is important to note that many palliative care needs can be appropriately assessed and effectively met with the support of general palliative care providers only. Developing sufficient skills and confidence among general palliative care providers to meet these growing needs requires strong leadership from specialist palliative care services.

Our findings add to a growing body of evidence demonstrating that high-quality, integrated care for older people with palliative care needs must include access to rehabilitation. A rehabilitative approach enables people with life-limiting illness to live as independently as possible, with choice and autonomy within the limits of advancing illness by providing them with interdisciplinary input, such as occupational therapy, physiotherapy, and mental health services [35–37]. Rehabilitation interventions and approaches improve quality-of-life [38], reduce unmet psychological needs [39], improve physical function outcomes [40] and may be highly cost-effective [41, 42]. Nevertheless, rehabilitative services remain an underutilised and underdeveloped component of care across most countries. Addressing this gap should be a priority for all health systems as the rates of disability burden in older age are set to increase sharply. Service design should be informed by evidence of best practice, with recent studies indicating that interventions initiated in the ED and continued into other settings have tended to result in more favourable patient and health service outcomes [12].

Notably, a significant proportion of participants had substantial, unmet oral health needs. Previous studies have highlighted that the prevalence of oral health conditions amenable to treatment (e.g., dry mouth, mucositis, candidiasis) among people with life-limiting illness can be as high as 90% [43–45]. Left unaddressed, oral health needs often interfere with speech, make chewing and swallowing difficult and painful, and limit social participation [44]. Despite recognition of these needs and the importance of providing oral health care, current service models often hinder appropriate access. For example, oral health professionals are typically not a part of the multidisciplinary team providing specialist palliative care services. Few oral health care professionals have the expertise or confidence to meet the needs of this population [44]. Additionally, individuals requiring dental care often need to attend community-based practices, a process which may be challenging or unfeasible for frail, vulnerable people. There is also a notable lack of oral health care in palliative care policy and guidance documents. Future research should identify optimal strategies to address these barriers, promote interdisciplinary collaboration and scale-up exemplars of best practice.

### Strengths and limitations

This study has both strengths and limitations. A key strength is that we successfully identified and recruited people with serious illness in the acute hospital setting, despite the widely documented challenges associated with such studies. Although our sample size is modest, our findings demonstrate the feasibility of collecting outcomes data directly from older people with life-limiting illness in this context. Such data are essential for informing policy and planning against the backdrop of an ageing population with rapidly changing needs.

However, the generalisability of our findings to other settings may be limited by some notable factors. This sample represents a subset of acute admissions to Oxford University Hospital as recruitment was limited to one of the four hospital sites within the Trust and primarily during typical office hours. Although approximately 2,000 patients were screened for recruitment, it was not possible to systematically record details on those excluded from study or reasons for exclusion. Similarly, it was not possible to systematically record the reasons leading to approximately one-third of participants not completing the reassessment questionnaires. Additionally, this study aimed to recruit a similar population in phase one and phase two; however, the profile and palliative care needs of people presenting to ED changed dramatically in phase two with the onset of the COVID-19 pandemic. Similar trends have been previously reported [46].

The data in this study covers a relatively short timeframe with some overlap in the recall period for the IPOS measure, and therefore may not fully reflect changes in health-related quality of life and symptom burden experienced by participants. The 72-hour interval was largely guided by practical aspects of clinical palliative care delivery, particularly that focus on early assessment. Additionally, early reassessment also reduced the potential for study attrition due to death, clinical deterioration or discharge.

## Conclusion

This study has demonstrated that older people with poor prognosis who experience an unplanned hospital admission have much higher symptom burden compared with population norms, though some improvement was observed on all measures at follow-up. These data provide valuable descriptive information on health-related quality of life among a priority population in practice and policy and can be used in future research to identify suitable interventions and model their effects.

### Electronic supplementary material

Below is the link to the electronic supplementary material.

Supplementary Material 1

Supplementary Material 2

Supplementary Material 3

## Data Availability

The anonymised datasets supporting the conclusions of this manuscript are available upon reasonable request to the study team, via the corresponding author.

## References

[1] Etkind SN, Bone AE, Gomes B, Lovell N, Evans CJ, Higginson IJ (2017). How many people will need palliative care in 2040? Past trends, future projections and implications for services. BMC Med.

[2] Sleeman KE, de Brito M, Etkind S, Nkhoma K, Guo P, Higginson IJ (2019). The escalating global burden of serious health-related suffering: projections to 2060 by world regions, age groups, and health conditions. Lancet Glob Health.

[3] May P, Johnston BM, Normand C, Higginson IJ, Kenny RA, Ryan K (2019). Population-based palliative care planning in Ireland: how many people will live and die with serious illness to 2046?. HRB Open Res.

[4] Smith AK, McCarthy E, Weber E, Cenzer IS, Boscardin J, Fisher J (2012). Half of older americans seen in emergency department in last month of life; most admitted to hospital, and many die there. Health Aff Proj Hope.

[5] Clark D, Armstrong M, Allan A, Graham F, Carnon A, Isles C (2014). Imminence of death among hospital inpatients: prevalent cohort study. Palliat Med.

[6] Higginson IJ, Evans CJ, Grande G, Preston N, Morgan M, McCrone P (2013). Evaluating complex interventions in end of Life Care: the MORECare Statement on good practice generated by a synthesis of transparent expert consultations and systematic reviews. BMC Med.

[7] Temel JS, Greer JA, Muzikansky A, Gallagher ER, Admane S, Jackson VA (2010). Early palliative care for patients with metastatic non-small-cell lung cancer. N Engl J Med.

[8] Howie L, Peppercorn J (2013). Early palliative care in cancer treatment: rationale, evidence and clinical implications. Ther Adv Med Oncol.

[9] Haun MW, Estel S, Rücker G, Friederich HC, Villalobos M, Thomas M (2017). Early palliative care for adults with advanced cancer. Cochrane Database Syst Rev.

[10] Hsien Seow R, Sutradhar F, Burge K, McGrail, Dawn M, Guthrie B, Lawson (2021). End-of-life outcomes with or without early palliative care: a propensity score matched, population-based cancer cohort study. BMJ Open.

[11] NHS (England). Universal Principles for Advance Care Planning. 2022 [cited 2023 October 28]. Available from: https://www.england.nhs.uk/wp-content/uploads/2022/03/universal-principles-for-advance-care-planning.pdf.

[12] Preston L, van Oppen JD, Conroy SP, Ablard S, Buckley Woods H, Mason SM (2021). Improving outcomes for older people in the emergency department: a review of reviews. Emerg Med J EMJ.

[13] Normand C (2009). Measuring outcomes in Palliative Care: limitations of QALYs and the Road to PalYs. J Pain Symptom Manage.

[14] Round J (2012). Is a QALY still a QALY at the end of life?. J Health Econ.

[15] Wichmann AB, Goltstein LCMJ, Obihara NJ, Berendsen MR, Van Houdenhoven M, Morrison RS (2020). QALY-time: experts’ view on the use of the quality-adjusted life year in cost-effectiveness analysis in palliative care. BMC Health Serv Res.

[16] Johnston BM, Normand C, May P (2017). Economics of Palliative Care: measuring the full value of an intervention. J Palliat Med.

[17] Mathew C, Hsu AT, Prentice M, Lawlor P, Kyeremanteng K, Tanuseputro P (2020). Economic evaluations of palliative care models: a systematic review. Palliat Med.

[18] Cardona M, Lewis ET, Kristensen MR, Skjøt-Arkil H, Ekmann AA, Nygaard HH (2018). Predictive validity of the CriSTAL tool for short-term mortality in older people presenting at Emergency departments: a prospective study. Eur Geriatr Med.

[19] Cardona M, O’Sullivan M, Lewis ET, Turner RM, Garden F, Alkhouri H et al. Prospective Validation of a Checklist to Predict Short-term Death in Older Patients After Emergency Department Admission in Australia and Ireland. Shah MN, editor. Acad Emerg Med. 2019;26(6):610–20. 10.1111/acem.13664 PMC6619350 30428145

[20] Herdman M, Gudex C, Lloyd A, Janssen M, Kind P, Parkin D (2011). Development and preliminary testing of the new five-level version of EQ-5D (EQ-5D-5L). Qual Life Res Int J Qual Life Asp Treat Care Rehabil.

[21] Murtagh FE, Ramsenthaler C, Firth A, Groeneveld EI, Lovell N, Simon ST (2019). A brief, patient- and proxy-reported outcome measure in advanced illness: validity, reliability and responsiveness of the Integrated Palliative care Outcome Scale (IPOS). Palliat Med.

[22] EuroQol - a (1990). New facility for the measurement of health-related quality of life. Health Policy.

[23] Devlin NJ, Shah KK, Feng Y, Mulhern B, van Hout B (2018). Valuing health-related quality of life: an EQ-5D-5L value set for England. Health Econ.

[24] John JR, Tannous WK, Jones A (2020). Changes in health-related quality of life before and after a 12-month enhanced primary care model among chronically ill primary care patients in Australia. Health Qual Life Outcomes.

[25] McNamara S, Schneider PP, Love-Koh J, Doran T, Gutacker N (2023). Quality-adjusted life expectancy norms for the English Population. Value Health.

[26] Dzingina MD, McCrone P, Higginson IJ (2017). Does the EQ-5D capture the concerns measured by the palliative care outcome scale? Mapping the palliative care Outcome Scale onto the EQ-5D using statistical methods. Palliat Med.

[27] Sopina E, Chenoweth L, Luckett T, Agar M, Luscombe GM, Davidson PM (2019). Health-related quality of life in people with advanced dementia: a comparison of EQ-5D-5L and QUALID instruments. Qual Life Res.

[28] Borchert K, Jacob C, Wetzel N, Jänicke M, Eggers E, Sauer A (2020). Application study of the EQ-5D-5L in oncology: linking self-reported quality of life of patients with advanced or metastatic colorectal cancer to clinical data from a German tumor registry. Health Econ Rev.

[29] Coast J, Bailey C, Kinghorn P. Patient centered outcome measurement in health economics: beyond EQ-5D and the Quality-Adjusted Life-Year—where are we now? 2018. 2018;S249–52. 10.21037/apm.2018.03.18 29860853

[30] Roch C, Palzer J, Zetzl T, Störk S, Frantz S, Van Oorschot B (2020). Utility of the integrated palliative care outcome scale (IPOS): a cross-sectional study in hospitalised patients with heart failure. Eur J Cardiovasc Nurs.

[31] Nicholson C, Davies JM, George R, Smith B, Pace V, Harris L (2018). What are the main palliative care symptoms and concerns of older people with multimorbidity?—a comparative cross-sectional study using routinely collected phase of illness, Australia-modified Karnofsky Performance Status and Integrated Palliative Care Outcome Scale data. Ann Palliat Med.

[32] McClure NS, Sayah FA, Xie F, Luo N, Johnson JA (2017). Instrument-defined estimates of the minimally important difference for EQ-5D-5L index scores. Value Health.

[33] European Observatory on Health Systems and Policies, Normand C, May P, Johnston B, Cylus J. Health and social care near the end of life: can policies reduce costs and improve outcomes? World Health Organization. Regional Office for Europe; 2021 [cited 2023 Jul 10]. 21 p. Available from: https://apps.who.int/iris/handle/10665/349803.

[34] Wang DH, Heidt R (2021). Emergency Department Admission Triggers for Palliative Consultation May decrease length of stay and costs. J Palliat Med.

[35] Barawid E, Covarrubias N, Tribuzio B, Liao S (2015). The benefits of Rehabilitation for Palliative Care patients. Am J Hosp Palliat Med.

[36] Health Service Executive (2017). Palliative care services: three year development framework (2017–2019).

[37] Wittry SA, Lam NY, McNalley T (2018). The value of Rehabilitation Medicine for patients receiving Palliative Care. Am J Hosp Palliat Care.

[38] Nottelmann L, Groenvold M, Vejlgaard TB, Petersen MA, Jensen LH (2021). Early, integrated palliative rehabilitation improves quality of life of patients with newly diagnosed advanced cancer: the Pal-Rehab randomized controlled trial. Palliat Med.

[39] Jones L, Fitzgerald G, Leurent B, Round J, Eades J, Davis S (2013). Rehabilitation in advanced, progressive, recurrent cancer: a randomized controlled trial. J Pain Symptom Manage.

[40] Chasen MR, Feldstain A, Gravelle D, Macdonald N, Pereira J (2013). An interprofessional palliative care oncology rehabilitation program: effects on function and predictors of program completion. Curr Oncol Tor Ont.

[41] Silver JK, Baima J, Mayer RS (2013). Impairment-driven cancer rehabilitation: an essential component of quality care and survivorship. CA Cancer J Clin.

[42] Smith S, Brick A, O’Hara S, Normand C (2014). Evidence on the cost and cost-effectiveness of palliative care: a literature review. Palliat Med.

[43] Fischer DJ, Epstein JB, Yao Y, Wilkie DJ (2014). Oral health conditions affect functional and social activities of terminally ill cancer patients. Support Care Cancer off J Multinatl Assoc Support Care Cancer.

[44] Chen X, Kistler CE (2015). Oral Health Care for older adults with serious illness: when and how?. J Am Geriatr Soc.

[45] Kvalheim SF, Strand GV. A narrative of oral care in Palliative patients. Int J Environ Res Public Health. 2022;19(10). 10.3390/ijerph19106306 PMC9141643 35627842

[46] Tan AJ, Swartz J, Wilkins C, Grudzen C (2022). Leveraging Emergency Department Information Systems to address Palliative Care needs of ED patients during the COVID pandemic. Am J Hosp Palliat Care.

