# Supplementary material for: Primary data on symptom burden and quality of life among elderly patients at risk of dying during unplanned admissions to an NHS hospital: a cohort study using EuroQoL and the integrated palliative care outcome scale
Source: BMC Palliat Care. 2024 Feb 20;23:46. doi: 10.1186/s12904-024-01384-9 (PMC10877897; doi:10.1186/s12904-024-01384-9)

**Additional File 1a IPOS scores by item at baseline**


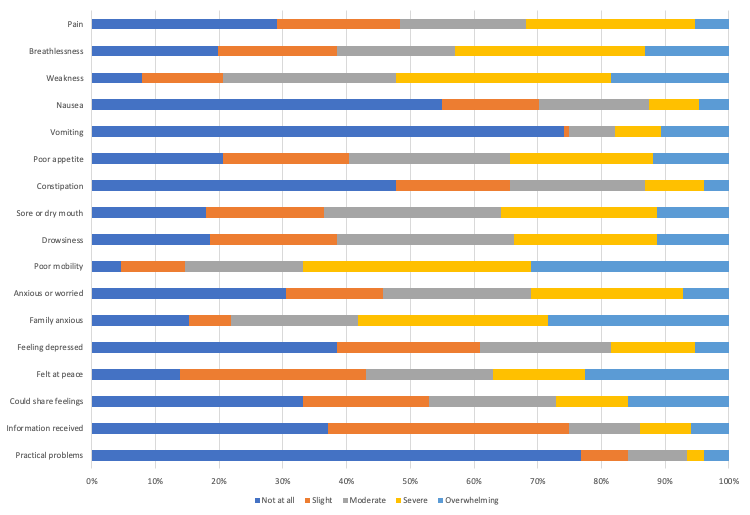


**Additional File 1b IPOS scores by item at reassessment**


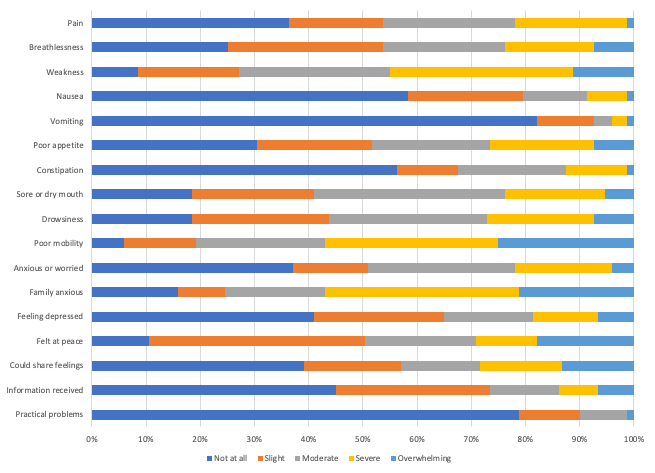

Supplement: Supplementary file 1 — Supplementary Material 1 [file 12904_2024_1384_MOESM1_ESM.docx]
