# Supplementary material for: Primary data on symptom burden and quality of life among elderly patients at risk of dying during unplanned admissions to an NHS hospital: a cohort study using EuroQoL and the integrated palliative care outcome scale
Source: BMC Palliat Care. 2024 Feb 20;23:46. doi: 10.1186/s12904-024-01384-9 (PMC10877897; doi:10.1186/s12904-024-01384-9)
Supplement: Supplementary file 2 — Supplementary Material 2 [file 12904_2024_1384_MOESM2_ESM.docx]

**Additional File 2 Correlation of EQ-5D-5L utility score, EQ VAS score and IPOS total score at baseline and follow up**


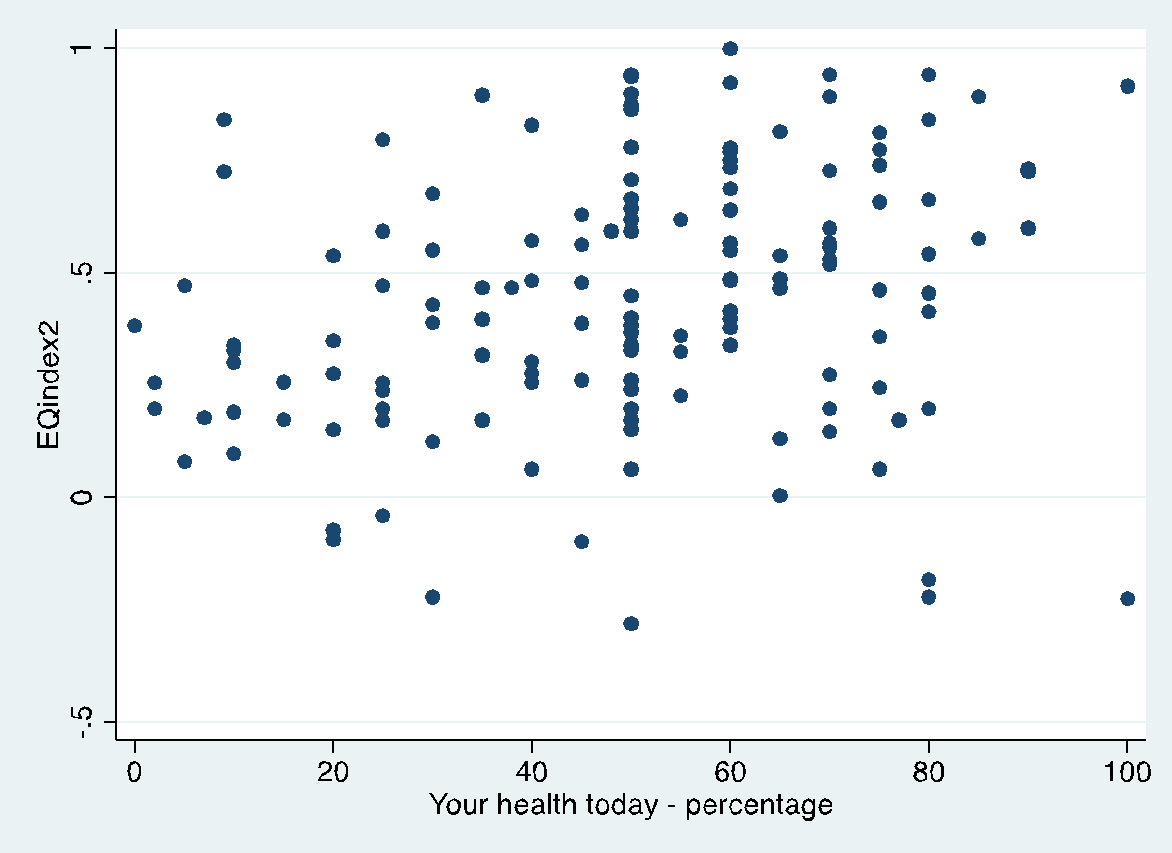

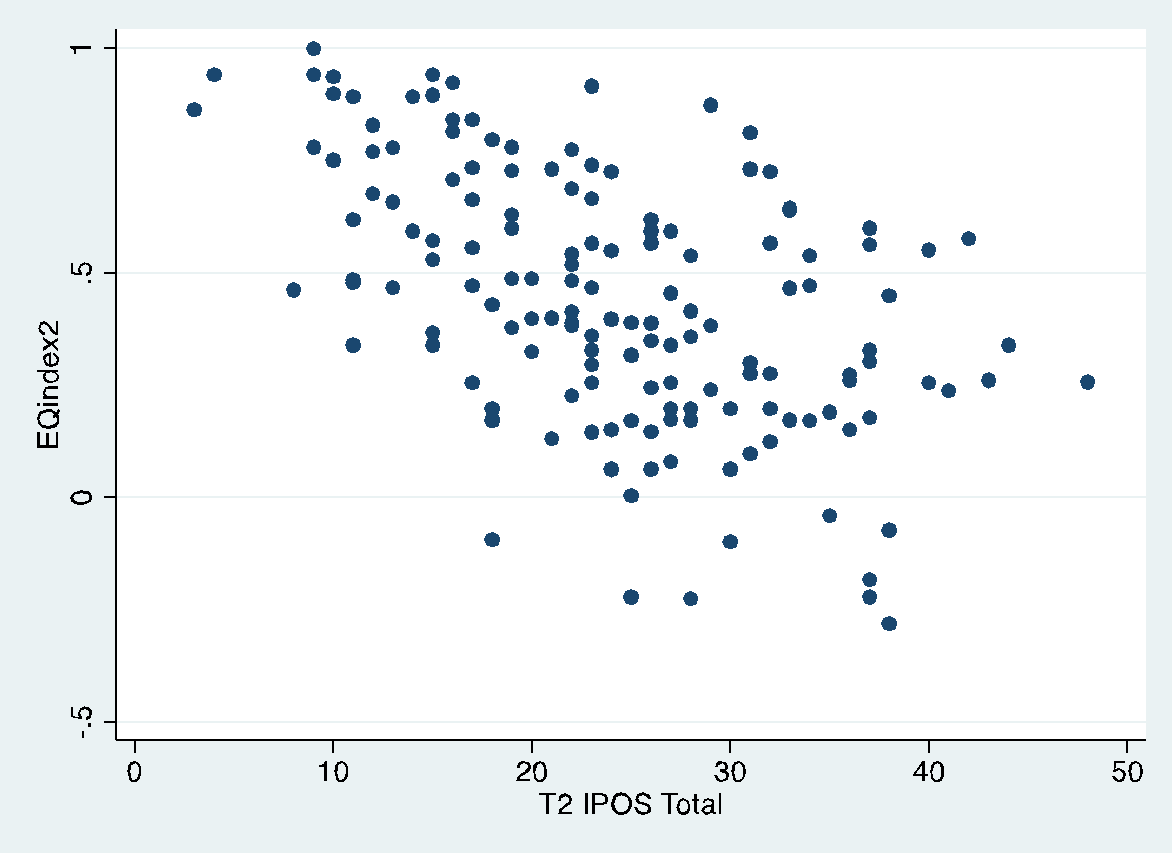

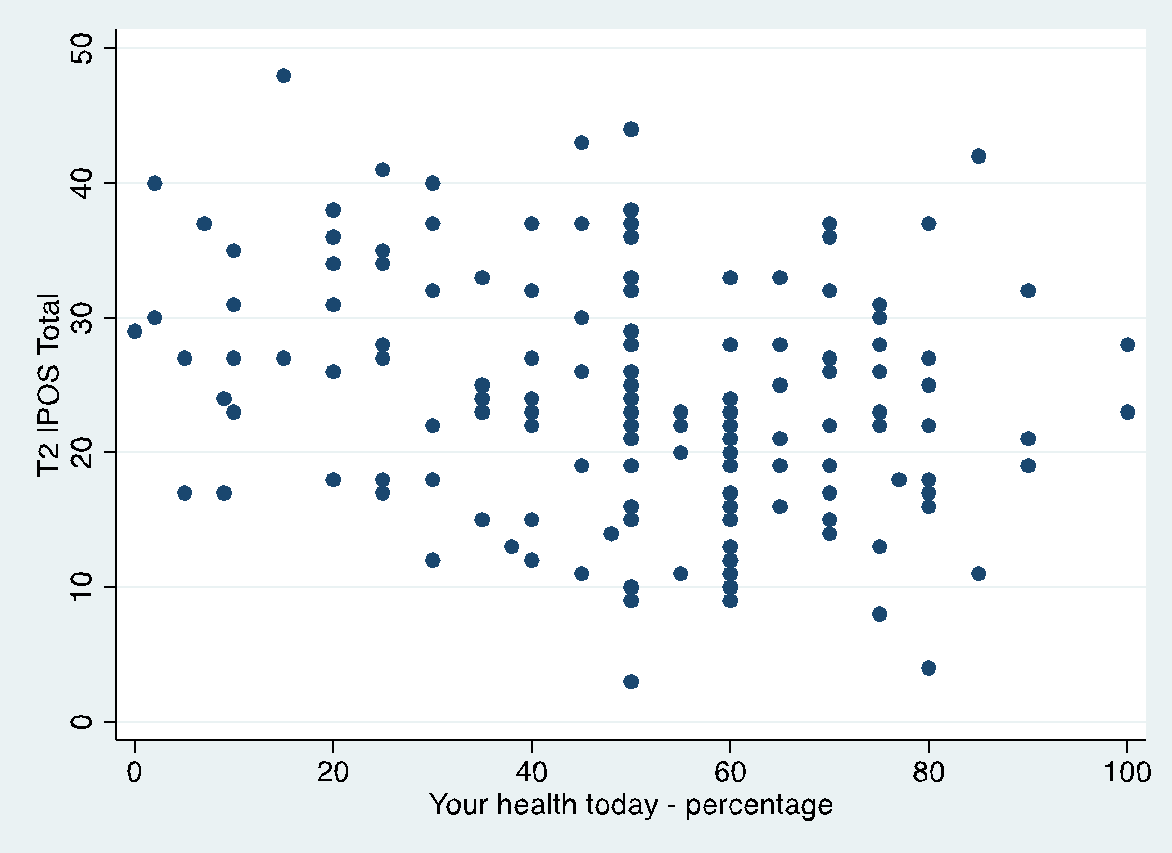


(ρ=-0.51, *p*<0.001)

(ρ=-0.27, *p*<0.001)

(ρ=-0.55, *p*<0.001)

(ρ=0.29, *p*<0.001)

(ρ=0.44, *p*<0.001)

(ρ=-0.54, *p*<0.001)
