# Supplementary material for: Primary data on symptom burden and quality of life among elderly patients at risk of dying during unplanned admissions to an NHS hospital: a cohort study using EuroQoL and the integrated palliative care outcome scale
Source: BMC Palliat Care. 2024 Feb 20;23:46. doi: 10.1186/s12904-024-01384-9 (PMC10877897; doi:10.1186/s12904-024-01384-9)
Supplement: Supplementary file 3 — Supplementary Material 3 [file 12904_2024_1384_MOESM3_ESM.docx]

Additional File 1 Participant characteristics (n=221)

| **Variable** | **Value** | **Missing data** |
| --- | --- | --- |
| **Gender: Female (%)** | 106 (48%) | 3 (1.4%) |
| **Age:** Median (IQR) | 84 (80 - 89) | 0 |
| **DNACPR in place** | 163 (73.8%) | 0 |
| **Marital status** |  | 18 (8.1%) |
| Single | 12 (5.4%) |  |
| Married | 84 (38.1%) |  |
| Divorced | 10 (4.5%) |  |
| Widowed | 97 (43.9%) |  |
| **CRiSTAL score** |  | 3 (1.4%) |
| 6 | 84 (38%) |  |
| 7 | 74 (33.5%) |  |
| 8 | 33 (14.9%) |  |
| 9 | 20 (9.1%) |  |
| 10 | 6 (2.7%) |  |
| 11 | 1 (0.5%) |  |
| **Charlson Index score** |  | 0 |
| 0 | 28 (12.7%) |  |
| 1 | 42 (19%) |  |
| 2 or higher | 151 (68.3%) |  |
| **Cancer** | 36 (16.3%) |  |
| **Deprivation** |  | 4 (1.8%) |
| 1 | 2 (0.9%) |  |
| 2 | 3 (1.4%) |  |
| 3 | 5 (2.3%) |  |
| 4 | 6 (2.7%) |  |
| 5 | 15 (6.8%) |  |
| 6 | 28 (12.7%) |  |
| 7 | 22 (10%) |  |
| 8 | 50 (22.6%) |  |
| 9 | 40 (18.1%) |  |
| 10 | 46 (20.8%) |  |
| **Known to department PC*** | 90 (40.7%) | 0 |
| **Length of stay:** median days (SD) | 6 (9) Range: 1-78 | 0 |
| **Died during index admission** | 27 (12.2%) | 0 |
| **Time from index admission to death:** median days (SD) | 56 (161.4) | 0 |
| **Readmissions in six months following the index admission:** Mean (Range) | 0.69 (0-8) | 0 |
| **ED reattendance in following six months** | 14 (6.3%) | 0 |
| **Died post discharge and within 6 months of enrolment**** | 63 (28.5%) | 0 |

*Known to PC: Had an interaction with departmental palliative care service prior to this admission

**Total deaths between admission and 6 months follow up = 90 (40.7% of study population)
